# Integrative analysis of a conceptual diagram depicting the relationship between pediatric ward nurses’ anxiety and physicians’ expectations when responding to sudden changes in children’s condition

**DOI:** 10.20407/fmj.2025-011

**Published:** 2026-02-28

**Authors:** Masato Sugiura, Masami Ishida, Ayumi Tasaki

**Affiliations:** Faculty of Maternal and Pediatric Nursing, School of Health Sciences, Fujita Health University, Toyoake, Aichi, Japan

**Keywords:** Pediatric ward nurses, Responding to sudden changes, Anxiety, Physicians’ expectations, Team-based care

## Abstract

**Objective:**

This study aimed to elucidate the relationship between pediatric ward nurses’ anxiety and physicians’ expectations when responding to sudden changes in children’s conditions by integrating findings from two previous qualitative studies.

**Methods:**

Core and higher-order categories extracted from two studies titled “Pediatric nurses’ anxiety about responding to sudden changes in children’s condition” and “Pediatricians’ expectations of pediatric nurses on sudden changes in children’s condition” were qualitatively synthesized and compared. The relationships among the categories were mapped and interpreted to identify convergences and divergences in emphasis.

**Results:**

Nurses’ anxiety about practical competence differed in nature from physicians’ expectations regarding organizational task execution, suggesting a contrast between nurses’ individual/affective concerns and physicians’ behavioral expectations. Anxiety related to patient/family engagement and environmental conditions aligned with physicians’ expectations, indicating shared priorities across all professions.

**Conclusions:**

The identified relationships underscore the importance of family centered care and interprofessional teamwork in pediatric ward settings. Targeted educational programs and organizational support are recommended to address differences in emphasis between nurses and physicians, and strengthened interprofessional understanding and collaboration are necessary to address the environmental conditions. An effective response to sudden changes in pediatric wards requires improving the practical competence of nurses and promoting team-based care grounded in mutual understanding with physicians.

## Introduction

Children admitted to pediatric wards are highly susceptible to rapid clinical deterioration due to their limited physiological reserves. Respiratory complications, which are a major cause of mortality in severe cases, necessitate particular vigilance when responding to sudden changes in children’s condition,^1^ hospitalization-related psychological stress further complicates their recovery process.^2^ As these vulnerabilities render pediatric patients prone to unexpected deterioration, pediatric ward nurses must respond both promptly and appropriately to sudden changes in the conditions of pediatric patients.

Effective management of sudden changes in children’s conditions relies on interprofessional teamwork, primarily between physicians and nurses. Rapid recognition of symptoms and timely interventions by nurses are crucial because they can directly influence patient outcomes.^3^ Effective interprofessional collaboration requires clear communication, mutual understanding, and psychological safety among team members,^4^ with interprofessional education and structured cooperation systems contributing to enhanced patient safety.^5^ Nevertheless, hierarchical dynamics and ambiguous role delineations often hinder effective communication between physicians and nurses,^6–8^ posing challenges in situations in which the children’s condition suddenly changes. These challenges are intricately linked to two key factors—namely, nurses’ anxiety and physicians’ expectations of nurses.” Nurses’ anxiety may delay reporting or suppress their opinions, whereas unclear or excessive physician expectations can create misunderstandings and obstruct smooth collaboration.

Qualitative investigations have separately evaluated nurses’ anxiety during pediatric emergencies^9^ and physicians’ expectations of nurses in such situations.^10^ However, nurses’ anxiety likely interacts reciprocally with physicians’ expectations, collectively influencing the quality of team communication and collaboration during sudden changes in children’s conditions. For instance, nurses’ anxiety about failing to meet physicians’ expectations may constrain their actions, whereas physicians’ lack of awareness about nurses’ anxiety, along with their excessive expectations, may exacerbate anxiety and further impair communication. A comprehensive understanding of this interrelationship is crucial for developing practical strategies aimed at improving communication and implementing educational approaches that promote mutual understanding—insights that cannot be obtained by examining anxiety and expectations in isolation. Additionally, elucidating this interrelationship may provide specific guidance for fostering more constructive communication between physicians and nurses. However, no research involving an integrative analysis of this relationship has been conducted.

This study aimed to elucidate the relationship between nurses’ anxiety and physicians’ expectations when responding to sudden changes in children’s conditions by conducting an integrative analysis of the findings from our two previous qualitative investigations. Through this analysis, this study sought to offer practical implications for advancing team-based care and improving interprofessional communication.

## Methods

### Definition of terms

#### Responding to sudden changes in children’s condition

Responding to sudden changes in children’s conditions was defined as the appropriate nursing actions performed immediately following the sudden deterioration of a pediatric patient’s clinical status until the provision of additional support. This encompassed suitable emergency procedures and nursing interventions performed in situations necessitating advanced urgent care, consistent with the definition of “assistance for people in critical health situations” by the Ministry of Education, Culture, Sports, Science and Technology^11^ and a qualitative studies.^9^

#### Anxiety

Conforming with the definitions by Herdman and Kamitsuru^12^ and Spielberger,^13^ anxiety was defined as an apprehensive feeling of often unknown origin that was triggered by perceived threats related to clinical deterioration.^9^ Anxiety as a personality trait and coping behaviors for reducing momentary anxiety were excluded based on a study.^9^

#### Expectation

Consistent with the definition in the seventh edition of Kōjien,^14^ expectation was defined as physicians’ reliance on nurses to achieve favorable outcomes when responding to sudden changes in children’s condition.^10^ This definition excluded anticipated performance levels and third-party evaluations.

### Study design

This qualitative secondary analysis integrated and reanalyzed existing data derived from two previously published qualitative studies to synthesize qualitative findings from the distinct viewpoints of nurses’ anxiety and physicians’ expectations when responding to sudden changes in children’s conditions and to elucidate challenges in team-based care.

### Target studies

Two qualitative studies titled “Pediatric nurses’ anxiety about responding to sudden changes in children’s condition”^9^ and “Pediatricians’ expectations of pediatric nurses on sudden changes in children’s condition”^10^ served as the analytical foundation. These two studies, conducted by the same research team and published in peer-reviewed journals, addressed different professional perspectives using qualitative methodologies. Additional domestic and international literature supplemented the background and discussion; however, no systematic review was performed.

Core categories extracted from each study are denoted as numbered circles (①–③) for anxiety-related categories and boxed numbers (1–4) for expectation-related categories. Anxiety-related core categories included “①Worries and distress associated with a sense of having inadequate skills to deal with sudden changes,” “②Concerns and distress about patients’ progress and their families’ reaction,” and “③Worries about disadvantageous environmental conditions and difficulties corresponding to these environmental conditions.” Expectation-related core categories included “1Ability to acquire knowledge that can facilitate anticipation of and response to changes in a patient’s condition,” “2Ability to perform all examinations and treatments and adjust to environmental conditions according to the growth and sudden changes in pediatric patients’ condition based on the doctor’s instructions and protocols,” “3Ability to communicate with the team during urgent situations,” and “4Ability to actively listen to complaints and explain the pediatric patient’s condition to family members.”

These core categories formed the foundation for the integrative analysis, and symbols for these categories were used consistently throughout the text, figures, and tables.

### Analysis methods and procedures

This qualitative secondary analysis integrated and interpreted core categories and conceptual diagrams from two previously published studies without collecting new data or conducting new analyses. The analysis followed five sequential steps: (i) organization of core and higher-order categories, (ii) comparison of conceptual diagrams, (iii) expert examination of relationships among categories, (iv) validation of interpretive validity through consensus building, and (v) development of a new, integrated conceptual diagram. The core and higher-order categories from the two studies were systematically organized, and conceptual diagrams were compared to identify structural similarities and differences. Categories representing identical phenomena or problem domains were interpreted as shared priorities common to both the professions.

Inter-rater agreement was confirmed by several researchers to ensure analytical rigor, with any discrepancies resolved through discussion. Expert consultation in qualitative research and pediatric nursing was sought to achieve consensus as necessary. The analysis was based exclusively on categories and diagrams from published studies, without reanalysis of the original interview data. Regular meetings were held by the research team to ensure interpretive consistency throughout key analytical phases. The final step involved integrating the findings into a novel conceptual diagram to visually depict the relationship between anxiety and expectations.

### Ethical considerations

This study builds upon a 2014 master’s thesis and two qualitative studies published in peer-reviewed journals of the Japan Society of Nursing Research and Japan Academy of Pediatric Nursing. Both original studies were approved by the institutional ethics review boards (approval no.: 14-101, approval date: May 15, 2014). Formal ethical approval from an ethics committee was not required because no new data were collected and only existing publications were analyzed. This qualitative secondary analysis used only published literature; thus, consent from research participants was not required.

The current integrative analysis adhered to the ethical guidelines. Multiple researchers, including subject-matter experts, verified the categories and conceptual diagrams to ensure interpretive validity and reliability. No conflicts of interest exist. The research process incorporated comprehensive supervision and discussions to maintain transparency and methodological rigor.

## Results

### Relationship between pediatric ward nurses’ anxiety and physicians’ expectations when responding to sudden changes in children’s condition

In this study, the core categories are presented as “anxiety-related” and “expectation-related,” with the specific labels placed in quotation marks. Analysis revealed several distinct relationships between pediatric ward nurses’ anxiety and physicians’ expectations (Table 1).

The most notable finding was that, with respect to shared priorities, the anxiety-related core category “①Worries and distress associated with a sense of having inadequate skills to deal with sudden changes” partially aligned with the expectation-related core categories “1Ability to acquire knowledge that can facilitate anticipation of and response to changes in a patient’s condition” and “2Ability to perform all examinations and treatments and adjust to environmental conditions according to the growth and sudden changes in pediatric patients’ condition based on the doctor’s instructions and protocols.” Furthermore, the anxiety-related core category “①Worries and distress associated with a sense of having inadequate skills to deal with sudden changes” shared areas of emphasis with the expectation-related core categories “1Ability to acquire knowledge that can facilitate anticipation of and response to changes in a patient’s condition” and “2Ability to perform all examinations and treatments and adjust to environmental conditions according to the growth and sudden changes in pediatric patients’ condition based on the doctor’s instructions and protocols.” This relationship suggests convergent priorities regarding knowledge, technical skills, and emotional aspects, whereas organizational and environmental factors showed divergent priorities (Table 2). The anxiety-related core category “①Worries and distress associated with a sense of having inadequate skills to deal with sudden changes” centered on personal competencies and experience, whereas the corresponding physicians’ expectations focused on broader task performance that encompassed organizational and environmental components, revealing differences in their respective priorities.

The anxiety-related core category “②Concerns and distress about patients’ progress and their families’ reaction” showed shared priorities with the expectation-related core categories “2Ability to perform all examinations and treatments and adjust to environmental conditions according to the growth and sudden changes in pediatric patients’ condition based on the doctor’s instructions and protocols” and “4Ability to actively listen to complaints and explain the pediatric patient’s condition to family members.” Similarly, the anxiety-related core category “③Worries about disadvantageous environmental conditions and difficulties corresponding to these environmental conditions” aligned with the expectation-related core categories “2Ability to perform all examinations and treatments and adjust to environmental conditions according to the growth and sudden changes in pediatric patients’ condition based on the doctor’s instructions and protocols” and “3Ability to communicate with the team during urgent situations.” These results indicate that both professions prioritize challenges related to patient and family care and team collaboration (Tables 3 and 4).

### Conceptual diagram of the relationship between anxiety and expectations

**Figure 1:**
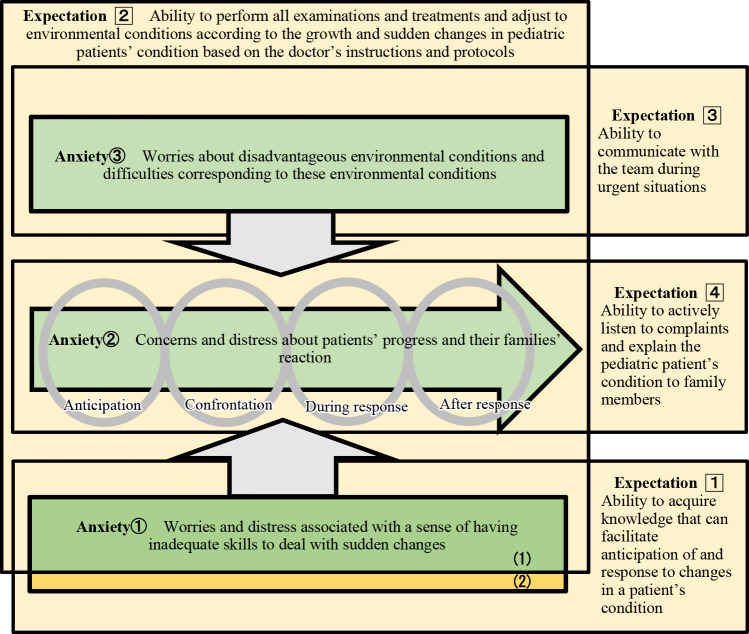
Conceptual diagram depicting the relationship between pediatric ward nurses’ anxiety and physicians’ expectations when responding to sudden changes. This diagram was created by integrating the findings of two previous studies. The four central circles represent “anticipation,” “confrontation,” “during response,” and “after response” and illustrate the chronological development of nurses’ anxiety. The diagram positions the anxiety-related core category “③Worries about disadvantageous environmental conditions and difficulties corresponding to these environmental conditions” at the top, anxiety-related core category “②Concerns and distress about patients’ progress and their families’ reaction” in the center, and anxiety-related core category “①Worries and distress associated with a sense of having inadequate skills to deal with sudden changes” at the bottom. On the right, the corresponding physicians’ expectations are represented: the expectation-related core category “1Ability to acquire knowledge that can facilitate anticipation of and response to changes in a patient’s condition,” expectation-related core category “2Ability to perform all examinations and treatments and adjust to environmental conditions according to the growth and sudden changes in pediatric patients’ condition based on the doctor’s instructions and protocols,” expectation-related core category “3Ability to communicate with the team during urgent situations,” and expectation-related core category “4Ability to actively listen to complaints and explain the pediatric patient’s condition to family members.” This integrative diagram visually illustrates the overlapping and distinct emphases between nurses’ anxiety and physicians’ expectations. The anxiety-related core category “①Worries and distress associated with a sense of having inadequate skills to deal with sudden changes” qualitatively shared key elements with the expectation-related core categories “1Ability to acquire knowledge that can facilitate anticipation of and response to changes in a patient’s condition” and “2Ability to perform all examinations and treatments and adjust to environmental conditions according to the growth and sudden changes in pediatric patients’ condition based on the doctor’s instructions and protocols.” This overlap was particularly evident in aspects related to practical competence tailored to the patient’s status and development **(1)**. In contrast, differences in emphasis were observed for task execution based on physicians’ instructions and for adjustment to the hospital environment **(2)**. *Notes*: **(1)** Common overlap mainly in the aspect of practical competence tailored to children’s conditions and developmental stages. **(2)** Aspect of task execution based on physicians’ instructions and adjustments to the ward environment. 1, 2: This partial overlap mainly occurred in the aspect related to practical competence tailored to children’s condition and developmental stage **(1)**, whereas differences in emphasis were evident for task execution based on physicians’ instructions and adjustment to the ward environment **(2)**. 3: In the figure, numbered circles (①–③) denote anxiety-related core categories, whereas boxed numbers (1–4) denote expectation-related core categories. These symbols are used consistently throughout the main text and in other tables of the manuscript.

A conceptual diagram (Figure 1) was developed based on these analyses to visually illustrate the relationship between nurses’ anxiety and physicians’ expectations. As depicted in the conceptual diagram, the anxiety-related core category “①Worries and distress associated with a sense of having inadequate skills to deal with sudden changes” exhibited a connection with the expectation-related core categories “1Ability to acquire knowledge that can facilitate anticipation of and response to changes in a patient’s condition” and “2Ability to perform all examinations and treatments and adjust to environmental conditions according to the growth and sudden changes in pediatric patients’ condition based on the doctor’s instructions and protocols,” with a shared emphasis on knowledge and technical skills while maintaining divergent priorities regarding organizational factors. The anxiety-related core categories “②Concerns and distress about patients’ progress and their families’ reaction” and “③Worries about disadvantageous environmental conditions and difficulties corresponding to these environmental conditions” showed shared priorities with the expectation-related core categories “2Ability to perform all examinations and treatments and adjust to environmental conditions according to the growth and sudden changes in pediatric patients’ condition based on the doctor’s instructions and protocols,” “3Ability to communicate with the team during urgent situations,” and “4Ability to actively listen to complaints and explain the pediatric patient’s condition to family members.”

A particularly noteworthy finding was that the expectation-related core category “2Ability to perform all examinations and treatments and adjust to environmental conditions according to the growth and sudden changes in pediatric patients’ condition based on the doctor’s instructions and protocols” was related to all the anxiety categories. Nevertheless, certain elements of physicians’ expectations had divergent priorities, reflecting fundamental differences in what nurses and physicians emphasize.

## Discussion

### Interpretation and significance of the relationship between anxiety and expectations

This study elucidated the relationship between pediatric ward nurses’ anxiety and physicians’ expectations of nurses when responding to sudden changes in children’s conditions. The analysis revealed convergent priorities between the anxiety-related core category “①Worries and distress associated with a sense of having inadequate skills to deal with sudden changes” and the expectation-related core categories “1Ability to acquire knowledge that can facilitate anticipation of and response to changes in a patient’s condition” and “2Ability to perform all examinations and treatments and adjust to environmental conditions according to the growth and sudden changes in pediatric patients’ condition based on the doctor’s instructions and protocols.” Nurses’ anxiety primarily reflected emotional responses to perceived deficiencies in personal knowledge, skills, or experience; in contrast, physicians emphasized rapid knowledge acquisition, practical skill improvement, and organizational task execution. This partial convergence indicates differing focal levels: individual concerns among nurses and organizational priorities among physicians.

In this study, the anxiety-related core categories “②Concerns and distress about patients’ progress and their families’ reaction” and “③Worries about disadvantageous environmental conditions and difficulties corresponding to these environmental conditions” aligned with corresponding physicians’ expectations. Family-related anxiety, which encompassed communication concerns, provision of information, and decisional support, paralleled the expectation-related core category “4Ability to actively listen to complaints and explain the pediatric patient’s condition to family members.” Environment-related anxiety, which encompassed staffing shortages, resource inadequacies, and team coordination deficits, aligned with the expectation-related core category “3Ability to communicate with the team during urgent situations.” These findings suggest a shared recognition of pediatric ward-specific challenges in both professions.

These results demonstrate how nurses’ anxiety and physicians’ behavioral expectations manifested as differing priorities while responding to sudden changes. A previous study reported distinct occupational values and priorities between nurses and physicians: nurses typically emphasize patient-centered care and emotional support, whereas physicians focus on clinical outcomes and decision-making processes.^15^ This study revealed how profession-specific perspectives materialized as both convergent and divergent priorities within the relationship between anxiety and expectations.

### Underlying factors and potential impact

The identified relationship between anxiety and expectations may have important implications for the quality of pediatric care. Two salient dimensions emerged: profession-specific perspectives and organizational factors. Nurses focused on patient-centered care and emotional support, whereas physicians tended to prioritize organizational task execution and decision making. This divergence was reflected in the mismatch between physicians’ expectations for organizational task execution and nurses’ anxiety regarding personal practical competence. Conversely, the convergence between family-related anxiety and the expectation-related core category “4Ability to actively listen to complaints and explain the pediatric patient’s condition to family members”) reflects the recognized importance of family-centered care in pediatric wards.^16^

With respect to organizational factors, the mismatch between physicians’ emphasis on organizational task execution and nurses’ anxiety about their individual practical competence may constitute a stressor in pediatric emergency nursing and potentially hinder effective team responses to clinical deterioration.^17^ This challenge may be further compounded by the authority imbalance between nurses and physicians in clinical settings, which can create barriers to effective collaboration and communication, potentially compromising patient care.^18^

### Practice implications

Considering the identified relationship between anxiety and expectations, integrated strategies are essential across three domains—namely, educational support, organizational measures, and communication enhancement.

### Educational support

In this study, the anxiety-related core category “①Worries and distress associated with a sense of having inadequate skills to deal with sudden changes” partially aligned with the expectation-related core categories “1Ability to acquire knowledge that can facilitate anticipation of and response to changes in a patient’s condition” and “2Ability to perform all examinations and treatments and adjust to environmental conditions according to the growth and sudden changes in pediatric patients’ condition based on the doctor’s instructions and protocols.” Given this finding, educational approaches should augment Benner’s novice-to-expert framework with explicit interprofessional collaboration elements.^19^ Competency-based curricula should include basic responses to sudden changes in procedures for novices, clinical judgment development via case discussions and simulation for intermediates, and leadership strengthening through team coordination training and interprofessional exercises for experts.^19^

### Organizational measures

The anxiety-related core category “③Worries about disadvantageous environmental conditions and difficulties corresponding to these environmental conditions” aligned with the expectation-related core category “3Ability to communicate with the team during urgent situations,” suggesting that role clarification and sharing, rather than merely increased staffing, are important. Interventions should prioritize information flow and family engagement while improving communication within the team.^20^ Organizational frameworks based on clear role delineation and shared accountability are recommended.^21^

### Communication enhancement

The relationship between the anxiety-related core category “③Worries about disadvantageous environmental conditions and difficulties corresponding to these environmental conditions” and the expectation-related core category “3Ability to communicate with the team during urgent situations” underscores the need for strengthened teamwork, role clarification, and designated leadership during emergencies.^22^ Joint simulation training and structured debriefing sessions for physicians and nurses can facilitate the identification of differences in role perceptions and shared challenges, thereby strengthening practical collaboration.^23^

These three domains are interconnected and require integrated implementation for optimal results. Practices informed by the identified relationship between anxiety and expectations may diminish nurses’ anxiety when responding to sudden changes, enhance their ability to meet physicians’ expectations, and ultimately improve patient safety and care quality.

### Limitations and future directions

The conceptual diagram created in this study visually depicts the relationship between nurses’ anxiety and physicians’ expectations in pediatric wards. However, this study had certain limitations. More than a decade has elapsed since the data were collected, and changes in clinical practice and nursing education may have altered the current realities. Additionally, the underlying qualitative studies were conducted at a single institution, consequently limiting the generalizability of the findings.

Future studies should incorporate quantitative validation across multiple institutions and more detailed analyses that consider factors such as nurses’ experience levels and physicians’ specialty areas. The identified divergence between nurses’ anxiety about individual practical competence and physicians’ expectations for organizational task execution suggests opportunities for developing targeted educational interventions, including simulation-based training and interprofessional collaboration programs. Addressing these gaps may improve mutual understanding in pediatric wards when responding to sudden changes and support the development of more effective educational frameworks.

## Conclusion

This study integrated findings from two qualitative studies and synthesized them into a comprehensive conceptual diagram to elucidate the relationship between pediatric nurses’ anxiety and physicians’ expectations when responding to sudden changes. Key findings indicated divergent priorities: nurses’ anxiety about individual practical competence versus physicians’ emphasis on organizational task execution. This divergence highlights the contrast between individual-level concerns and organizational-level expectations and reflects the differing focal points between nurses’ emotional factors and physicians’ behavioral expectations.

Anxiety related to patient and family care and environmental conditions aligned with the corresponding physician’s expectations, indicating a shared recognition of these challenges. This suggests that multifaceted approaches encompassing knowledge and skill enhancement, ongoing educational programs, organizational initiatives fostering interprofessional understanding, and comprehensive strategies addressing family centered care and environmental factors are necessary to improve the quality of responses to sudden changes in children’s conditions in pediatric wards.

The developed conceptual diagram provides a foundation for understanding the relationship between nurses’ anxiety and physicians’ expectations and differences in priorities, offering a framework for developing targeted interventions to enhance the quality of response to sudden changes in children’s conditions in pediatric wards.

## Figures and Tables

**Table 1:** Relationship between core categories related to pediatric ward nurses’ anxiety and physicians’ expectations during an emergency response

Core categories of nurses’ anxiety		Core categories of physicians’ expectations	
①	Worries and distress associated with a sense of having inadequate skills to deal with sudden changes	1	Ability to acquire knowledge that can facilitate anticipation of and response to changes in a patient’s condition”
		2	Ability to perform all examinations and treatments and adjust to environmental conditions according to the growth and sudden changes in pediatric patients’ condition based on the doctor’s instructions and protocols
②	Concerns and distress about patients’ progress and their families’ reaction	2	Ability to perform all examinations and treatments and adjust to environmental conditions according to the growth and sudden changes in pediatric patients’ condition based on the doctor’s instructions and protocols
		4	Ability to actively listen to complaints and explain the pediatric patient’s condition to family members
③	Worries about disadvantageous environmental conditions and difficulties corresponding to these environmental conditions	2	Ability to perform all examinations and treatments and adjust to environmental conditions according to the growth and sudden changes in pediatric patients’ condition based on the doctor’s instructions and protocols
		3	Ability to communicate with the team during urgent situations

In the tables, numbered circles (①–③) denote anxiety-related core categories, whereas boxed numbers (1–4) denote expectation-related core categories. These symbols are used consistently throughout the main text and in other tables.

**Table 2:** Relationship between anxiety ① (higher-order categories) and expectations (subcategories)

Anxiety-related core categories	Higher-order categories of anxiety		Subcategories of expectations	Core categories of physicians’ expectations
①Worries and distress associated with a sense of having inadequate skills to deal with sudden changes	1	Upset or difficulty due to lack of experience in responding to sudden changes	1, 2	1
			3, 4, 5	2
	2	Anxiety about insufficient knowledge for responding to sudden changes according to the child’s age and development	1, 2	1
			3, 4, 5	2
	3	Concern about being held responsible because of inadequate judgment during sudden changes	1, 2	1
			3, 4, 5	2
	4	Worry or unease about insufficient technical skills for responding to sudden changes according to the child’s age and development	1, 2	1
			3, 4, 5	2
	5	Difficulty in appropriate response due to lack of emotional control	2	1
			4, 5	2

The subcategories of expectations (numbered in the table) are defined as follows: 1. Can acquire basic knowledge to assess the child’s condition 2. Can anticipate and respond appropriately to physician instructions 3. Can prepare and use equipment and medications suitable for the child’s condition and development 4. Can efficiently assist with examinations, treatments, and procedures based on the child’s condition 5. Can perform life-sustaining techniques The core categories of physicians’ expectations (numbered in the table) are defined as follows: 1Ability to acquire knowledge that can facilitate anticipation of and response to changes in a patient’s condition 2Ability to perform all examinations and treatments and adjust to environmental conditions according to the growth and sudden changes in pediatric patients’ condition based on the doctor’s instructions and protocols

**Table 3:** Relationship between anxiety ② (higher-order categories) and expectations (subcategories)

Anxiety-related core categories	Higher-order categories of anxiety		Subcategories of expectations	Core categories of physicians’ expectations
②Concerns and distress about patients’ progress and their families’ reaction	6	Vigilance toward anticipated patient deterioration	4, 5, 6, 7	2
	7	Distress or apprehension when facing sudden changes	5, 7	2
	8	Regret after the occurrence of sudden deterioration	5, 7	2
	9	Fear or anxiety about worsening condition due to inadequate response	3, 4, 5, 6, 7	2
	10	Impatience about lack of recovery	4, 5, 6	2
	11	Concern about being held responsible	4, 5, 6, 7	2
	12	Anxiety or fear over unstable condition and potential life-threatening outcomes after responding to sudden changes	4, 5, 6	2
	13	Fear regarding unexpected progression to severe illness	5, 6, 7	2
	14	Anxiety about sequelae and recognition of accountability	5, 6	2
	15	Alarm or distress about heightened family anxiety during sudden changes	12	4
	16	Difficulty relating to the feelings of the family	12	4

Subcategories of expectations (numbered in the table) are defined as follows: 3. Can prepare and use equipment and medications suitable for the child’s condition and development 4. Can efficiently assist with examinations, treatments, and procedures based on the child’s condition 5. Can perform life-sustaining techniques 6. Can fully implement all physician instructions 7. Can organize and maintain the bedside environment for the child 12. Can listen to family concerns and explain the child’s situation and progress to alleviate anxiety Core categories of physicians’ expectations (numbered in the table) are defined as follows: 2Ability to perform all examinations and treatments and adjust to environmental conditions according to the growth and sudden changes in pediatric patients’ condition based on the doctor’s instructions and protocols 4Ability to actively listen to complaints and explain the pediatric patient’s condition to family members

**Table 4:** Relationship between anxiety ③ (higher-order categories) and expectations (subcategories)

Anxiety-related core categories	Higher-order categories of anxiety		Subcategories of expectations	Core categories of physicians’ expectations
③Worries about disadvantageous environmental conditions and difficulties corresponding to these environmental conditions	17	Frustration or difficulty in responding to sudden changes due to inadequate collaboration with physicians	3, 4, 5, 6, 7	2
			8, 9, 10, 11	3
	18	Difficulty in role allocation among nurse team members	3, 4, 5, 6, 7	2
			8, 9, 10, 11	3
	19	Anxiety or impatience regarding inadequate response to the child’s deterioration due to staff shortages	3, 4, 5, 6, 7	2
			8, 9, 10, 11	3
	20	Confusion or impatience about changes in the child’s condition when only one nurse is available	3, 4, 5, 6, 7	2
			8	3
	21	Worry or confusion regarding insufficient response to other patients due to manpower shortage	8, 11	3
	22	Concern about delayed response because of a lack of necessary supplies for the child’s condition	3, 4, 5, 6, 7	2
	23	Worry or frustration about delays caused by insufficient or unusable supplies	3, 4, 5, 6, 7	2
	24	Concern about delayed response to sudden changes when the family is absent	6, 7	2
			8	3

Subcategories of expectations (numbered in the table) are defined as follows: 3. Can prepare and use equipment and medications suitable for the child’s condition and development 4. Can efficiently assist with examinations, treatments, and procedures based on the child’s condition 5. Can perform life-sustaining techniques 6. Can fully implement all physician instructions 7.Can organize and maintain the bedside environment for the child 8. Can secure sufficient staff and fulfill assigned roles 9. Can share information between physicians and nurses 10. Can communicate urgent and critical information 11. Can maintain a calm tone and foster a positive atmosphere Core categories of physicians’ expectations (numbered in the table) are defined as follows: 2Ability to perform all examinations and treatments and adjust to environmental conditions according to the growth and sudden changes in pediatric patients’ condition based on the doctor’s instructions and protocols 3Ability to communicate with the team during urgent situations

## Availability of Data and Materials

This study is a qualitative secondary analysis that exclusively integrated and reanalyzed data (core categories and conceptual diagrams) extracted from two previously published qualitative studies. No new data were collected or analyzed. All data supporting the findings of this study are available within the following published articles:

Sugiura M, Morita M, Hagimoto A. Pediatric Nurses’ Anxiety about Responding to Sudden Changes in Children’s Condition. Journal of Japan Society of Nursing Research 2019; 42: 735–47 (in Japanese).

Sugiura M. Pediatricians’ Expectations of Pediatric Nurses on Sudden Changes in Children’s Condition. Journal of Japanese Society of Child Health Nursing 2022; 31: 61–9 (in Japanese).

Any requests for detailed information, materials, or conceptual diagrams described in this manuscript will be reasonably considered.

## Supplementary Material

PDF-Japanese
