# Supplementary material for: Integrative analysis of a conceptual diagram depicting the relationship between pediatric ward nurses’ anxiety and physicians’ expectations when responding to sudden changes in children’s condition
Source: Fujita Med J. 2026 Feb 28;12(2):121–8. doi: 10.20407/fmj.2025-011 (PMC13129720; doi:10.20407/fmj.2025-011)
Supplement: Supplementary file 1 — PDF-Japanese [file fmj-12-121_s1.pdf]

**タイトルページ**

**タイトル：**

小児病棟看護師の急変対応における不安と医師の期待の関連性に関する概念図の統合分析

**ランニングタイトル（140 字以内）：**

小児病棟急変対応における看護師の不安と医師の期待

**全著者（フルネーム）学位（MD，PhD 等），所属：**


**・Masato Sugiura, MS, Faculty of Maternal and Pediatric Nursing, School of Health Sciences, Fujita Health University, Toyoake, Aichi, Japan**

**・Masami Ishida, MS, Faculty of Maternal and Pediatric Nursing, School of Health Sciences, Fujita Health University, Toyoake, Aichi, Japan**

**・Ayumi Tasaki, PhD, Faculty of Maternal and Pediatric Nursing, School of Health Sciences, Fujita Health University, Toyoake, Aichi, Japan**


**論文の種類：**

原著 Original Article

**Corresponding author，連絡先住所，電話番号・メールアドレス：**


Masato Sugiura，MS,

Faculty of Maternal and Pediatric Nursing, School of Health Sciences, Fujita Health University,

1-98, Dengakugakubo, Kutsukake-cho, Toyoake, Aichi 470-1192, Japan

Telephone: 0562-93-2598; Email: s-mas@fujita-hu.ac.jp

**本文**

# 小児病棟看護師の急変対応における不安と医師の期待の関連性に関する概念図の統合分析

**抄録**：

目的：小児病棟看護師の急変対応における不安と医師の期待との関連性を，著者らの先行する 2 つの質的研究結果を統合分析することで明らかにした。方法：「小児病棟看護師の急変対応に関する不安」と「小児病棟看護師の急変対応における医師の期待」の 2 つの質的研究から抽出されたコアカテゴリーと上位カテゴリーの関連性を質的に統合・比較した。結果：看護師の実践能力に関する不安は，医師が期待する組織的な業務遂行能力とは性質が異なり，看護師の個人的・感情的要因と医師の行動的期待に重視する側面の違いが明らかとなった。一方，患児・家族への対応や環境条件に関する不安は，医師の期待と重視する側面が一致しており，両職種の共通課題として認識されていた。考察：不安と期待の関連性から，小児病棟における家族中心ケアやチーム医療の重要性が示唆された。看護師と医師が重視する側面の違いには教育プログラムや組織的支援による調整が必要であり，環境条件への対応には多職種間の相互理解と連携強化が重要である。結論：小児病棟での効果的な急変対応には，看護師の実践能力向上とともに，医師との相互理解に基づくチーム医療の推進が不可欠である。

**キーワード**:

小児病棟看護師，急変対応，不安，医師の期待，チーム医療

## 緒　言

小児病棟に入院する子どもは身体的予備能力が少なく，症状が急速に進行しやすい特徴がある。特に重症例では呼吸器疾患が重要な死亡要因となり，急変時の対応には特別な注意が必要である[1]。また，入院による心理的ストレスは子どもの回復過程を複雑化させる要因となる[2]。これらの

脆弱性により，小児患者の状態は予期せず急変しやすく，小児病棟看護師には迅速かつ適切な対応が求められる。

急変対応には，医師と看護師を中心としたチーム医療が不可欠であり，特に看護師の迅速な症状の察知と介入が患者の予後に大きく影響する[3]。効果的なチーム医療のためには明確なコミュニケーションが重要であり，職種間の相互理解や心理的安全性の構築が求められる[4]。さらに，多職種間の教育や協力体制も患者ケアの安全性の向上に寄与する[5]。しかし，医師と看護師の間には階層構造や役割の不明確さが存在し，コミュニケーションを阻害する要因となっている[6, 7, 8]。このような小児の急変時における医師と看護師間の課題は,「看護師が抱える不安」と「医師が看護師に寄せる期待」に深く関連している。看護師の不安は迅速な報告や意見表明をためらわせ，医師の期待が不明確な場合，認識の相違や円滑な連携を阻害する恐れがある。

著者らはこれまで，小児病棟看護師の急変対応における不安[9]と，医師が看護師に期待する内容[10]を質的に明らかにしてきた。しかし，看護師の不安と医師の期待は，互いに影響し合いながら，急変時のチームコミュニケーションや連携の質に作用していると考えられる。例えば，医師の期待に応えられないかもしれないという不安が看護師の行動を制限したり，逆に看護師の不安を理解したりしないまま過度な期待を寄せることが，さらなる不安やコミュニケーション不足につながる可能性がある。この両者の関連性を包括的に理解することは，単に個別の不安や期待を把握するだけでは得られない，より実践的なコミュニケーションの改善策や，相互理解を促進するための教育的アプローチを開発する上で極めて重要である。 この相互作用を解明することで，医師と看護師間のより建設的なコミュニケーションを促進するための具体的な示唆を得ることが期待できる。しかし両者の関連性を統合的に分析した研究は見当たらない。

そこで本研究は，小児病棟における急変対応時の看護師の不安と医師の期待の関連性を，既存の 2 つの質的研究の結果を統合・分析することで明らかにし，チーム医療やコミュニケーション改善への示唆を得ることを目的とした。

## 研究方法

### *A．用語の定義*

#### *1．急変対応*

本研究では，文部科学省[11]の「健康の危機的状況にある人への援助」に基づき，「急変した患児に応じて応援が到着するまでに行う救急処置や，高度な緊急治療が必要となる場面経過に応じた看護師の適切な言動」[9]を急変対応と定義する。

#### *2. 不安*

本研究では，Herdman & Kamitsuru[12]および Spielberger[13]の定義に基づき，「本人に原因は特定できないことが多く，急変という危険の予感によって生じる心配な感情」[9]とする。なお，先行研究[9]と同様に，性格特性としての不安や不安状態を軽減させようとして働く言動は対象外とした。

#### *3. 期待*

新村[14]の広辞苑第七版の定義に基づき，「看護師が急変対応でもたらすよい結果」を「医師がその実現を当てにすること全て」を期待と定義する。この定義は先行研究[10]と同一であり，同様に，どのくらいの成果をもたらすかという見込みや他者評価の内容は含めない。

### *B．研究デザイン*

本研究は，既発表の 2 本の質的研究論文の既存データを新たな視点で再分析・統合する質的二次分析研究である。看護師の不安と医師の期待という異なる視点から得られた質的成果を統合し，チーム医療の課題を明らかにするため，この研究デザインを選択した。

### *C．分析対象論文*

分析対象は「小児病棟看護師の急変対応における不安」[9]および「小児病棟看護師の急変対応における医師の期待」[10]の 2 本の質的研究論文である。いずれも同一研究者によるもので，看護師と医師の異なる視点を扱い，両論文とも質的研究手法を用いた査読付き学術誌掲載論文である。他の国内外文献は背景や考察の補強など補助的に参照したが，システマティックレビューは

行っていない。以下のそれぞれの 2 本の質的研究論文から抽出されたコアカテゴリーは【　】で示した。不安に関するコアカテゴリーには丸数字（①～③），医師の期待に関するコアカテゴリーには四角囲み数字（[1]～[4]）を使用している。

不安に関するコアカテゴリーは【①急変時の実践能力不足による心配や動揺】【②患児・家族の状態の経過から認識された危惧や動揺】【③不利な環境条件とその対応への心配や困難感】の 3 つ，医師の期待は【[1]患児の状況に合わせた予測や対応に関する知識を習得している】【[2]患児の状況・成長を考慮し医師の指示に基づいた処置・検査・治療の実施と環境調整ができる】【[3]緊急度に合わせたチームのコミュニケーションづくり】【[4]家族への傾聴や児の状況説明ができる】の 4 つのコアカテゴリーに整理されている。本研究では，これらのコアカテゴリーを統合分析の対象とし，記号は本文，図，表に一貫して用いた。

***D．分析方法と手順***

本研究は， 既存の 2 本の質的研究論文から抽出されたコアカテゴリーおよび概念図を統合・分析する単一の質的二次分析研究である。新たなデータ収集や分析は行わず，既存データを用いて関連性を明らかにした。

分析手順は，段階的に①コアカテゴリーと上位カテゴリー（サブカテゴリー）の整理，②概念図の比較，③専門家による関連性の検討，④合意形成による解釈の妥当性の確認，⑤新たな概念図の作成，という流れで進めた。まず，両論文から抽出されたコアカテゴリー，上位カテゴリー（サブカテゴリー）を体系的に整理し，概念図を比較して構造的な類似点と相違点を明確にした。カテゴリーが同一の現象や課題領域を指す場合は「重視する側面が共通している」と判断し，両職種の共通課題として解釈した。分析の信頼性を確保するため，複数の研究者間で解釈の一致度を確認し，相違点は議論を再検討した。必要に応じて質的研究や小児看護学の専門家に意見を求め，合意形成を図った。原データ（インタビュー記録や逐語録等）は再分析せず，既存論文で抽出されたカテゴリーや概念図を分析対象とした。分析の主要な節目ごとに研究者間でミーティングを重ね，解釈の一貫性を確保した。最終的に，これらの分析結果を統合し，不安と期待の相互

の関連性を視覚的に表現した新たな概念図を作成した。

### *D．倫理的配慮*

本研究は，2014 年度に提出した修士論文および日本看護研究学会，日本小児看護学会誌に掲載された 2 本の質的研究成果を基に，不安と期待の関連性を新たな視点で統合・分析した。両研究は所属機関の倫理審査委員会の承認（受付番号：14-101，承認日：2014 年 5 月 15 日）を得て実施されたものである。今回の質的統合分析も倫理規程を遵守し，専門家を含む複数名でカテゴリーや概念図の確認を行い，解釈の妥当性や信頼性を確保した。利益相反は存在せず，研究の透明性と信頼性を高めるため，スーパーバイズを含む十分な検討プロセスを経て進められた。

## 研究結果

### *A．急変時に小児病棟看護師が抱く不安と医師の期待との関係*

本研究における不安のコアカテゴリーは【　】(以下,「不安【　】」)，上位カテゴリーは『　』で示す。同様に，医師の期待のコアカテゴリーは【　】(以下,「期待【　】」)，サブカテゴリーは[　]で示す。なお，方法で述べたように，不安のコアカテゴリーには丸数字（①～③)，医師の期待のコアカテゴリーには四角囲み数字（1～4）を用いている。分析の結果，小児病棟看護師が抱く不安と医師の期待との間にいくつかの特徴的な関連性が見出された（**表 1**）。

最も特徴的な点は，看護師の不安【①急変時の実践能力不足による心配や動揺】は，医師の期待【1患児の状況に合わせた予測や対応に関する知識を習得している】および【2患児の状況・成長を考慮し医師の指示に基づいた処置・検査・治療の実施と環境調整ができる】と重視する側面が一部共通している点である。この関連性は，知識・技術・感情面では重視する側面が共通している一方で，組織的・環境的要因については重視する側面が一致しないことが観察された（**表 2**）。すなわち，看護師の不安【①急変時の実践能力不足による心配や動揺】が個人の能力や経験に関する不安を示しているのに対し，これに関連する医師の期待は組織的・環境的な要素を含む業務遂行に焦点を当てており，重視する側面に違いが見られた。

対照的に，看護師の不安【②患児・家族の状態の経過から認識された危惧や動揺】は，医師の期待【2患児の状況・成長を考慮し医師の指示に基づいた処置・検査・治療の実施と環境調整ができる】および【4家族への傾聴や児の状況説明ができる】と重視する側面が共通していることが質的に明らかになった。同様に，看護師の不安【③不利な環境条件とその対応への心配や困難感】は，医師の期待【2患児の状況・成長を考慮し医師の指示に基づいた処置・検査・治療の実施と環境調整ができる】および【3緊急度に合わせたチームのコミュニケーションづくり】と重視する側面が一致することが確認された。これらは，患児や家族への対応やチーム連携といった課題が，両職種で重視する側面が共通していることを反映していた（**表３・表４**）。

### *B．コアカテゴリーによる不安と期待の関係に関する概念図*

これらの分析結果を基に，小児病棟看護師が抱く不安と医師が看護師に期待する内容との関連性を明らかにし，それを視覚的に表現した概念図（**図１**）を作成した。この図では，看護師の不安【①急変時の実践能力不足による心配や動揺】と医師の期待【1患児の状況に合わせた予測や対応に関する知識を習得している】【2患児の状況・成長を考慮し医師の指示に基づいた処置・検査・治療の実施と環境調整ができる】の間には，知識・技術面で重視する側面が一致し，組織的要素では重視する側面が異なることが示された。一方，看護師の不安【②患児・家族の状態の経過から認識された危惧や動揺】【③不利な環境条件とその対応への心配や困難感】は，医師の期待【2患児の状況・成長を考慮し医師の指示に基づいた処置・検査・治療の実施と環境調整ができる】【3緊急度に合わせたチームのコミュニケーションづくり】【4家族への傾聴や児の状況説明ができる】と重視する側面が共通していることが明らかになった。

特に，医師の期待【2患児の状況・成長を考慮し医師の指示に基づいた処置・検査・治療の実施と環境調整ができる】はすべての不安のカテゴリーと関連するが，医師の期待の一部の要素では重視する側面が一致せず，両者の重視する側面の違いを反映していた。

## 考察

### *A. 不安と期待の関連性の解釈とその意義*

本研究では，小児病棟看護師が抱く不安と医師が看護師に期待する内容の関連性を質的に分析した。その結果，看護師の不安【①急変時の実践能力不足による心配や動揺】は，医師の期待【1患児の状況に合わせた予測や対応に関する知識を習得している】【2患児の状況・成長を考慮し医師の指示に基づいた処置・検査・治療の実施と環境調整ができる】と，重視する側面が一部共通していることが質的に確認された。看護師の不安【①急変時の実践能力不足による心配や動揺】は，看護師個人の知識・技術・経験不足による感情的要因が主であり，一方で医師は知識習得と迅速な実践能力向上，組織的な業務遂行能力を期待していた。この重視する側面が一部共通している点は，個人レベルと組織レベルで焦点が異なることを反映している。

看護師の不安【②患児・家族の状態の経過から認識された危惧や動揺】および【③不利な環境条件とその対応への心配や困難感】については，それぞれ医師の期待と重視する側面が一致していることが質的に確認された。不安【②患児・家族の状態の経過から認識された危惧や動揺】では，家族への情報提供や意思決定支援など家族対応への不安が顕著であり，医師も同様に【4家族への傾聴や児の状況説明ができる】ことを期待していた。看護師の不安【③不利な環境条件とその対応への心配や困難感】については，マンパワー不足，物品準備不足，チーム連携不足などから構成され，医師は【3緊急度に合わせたチームコミュニケーションづくり】を期待しており，これらが小児病棟特有の課題として両職種で共通認識されていることを示している。

これらの分析結果は，看護師が抱える感情的な不安と医師から求められる行動的な期待が，急変対応時に重視する側面（視点）の違いとして現れていることを明らかにした。先行研究では，医師と看護師では職業的価値観と優先事項に明確な違いが見られ，看護師は患者中心のケアと感情的サポートを重視する傾向がある一方，医師は臨床結果と意思決定プロセスに焦点を当てることが多いと報告されている[15]。本研究では，この職種間の視点の違いが，不安と期待が一部の重視する側面で共通していたことや，特定の要素においてそれぞれ異なる側面に焦点が当てられていることとして具体化されていると考えられた。

### *B. 関連性の生じる背景要因およびその影響*

本研究で明らかになった不安と期待の関連性は，小児医療の質に重要な影響を与える可能性があり，その背景には職種特有の視点と組織的要因という二つの側面が存在すると考えられる。看護師が患者中心のケアと感情的サポートに重視する一方で，医師は組織的な業務遂行や意思決定を重視する傾向がある。この違いは，医師の期待に含まれる組織的な業務遂行に関する要素と，看護師の個人的な実践能力不足による不安との間に焦点を当てる側面の違いとして表れている。これに対し，家族対応への不安と医師の期待【4家族への傾聴や児の状況説明ができる】の重視する側面が一致している点は，小児病棟で家族中心ケアが重要視されていることを反映している[16]。

組織的要因については，医師が組織的な業務遂行能力を重視する一方で，看護師が個人の実践能力不足に不安を抱えるという視点の違いは，小児救急看護におけるストレス要因となり，チーム全体としての効果的な急変対応を妨げる要因となり得る[17]。この背景には，医療現場における看護師と医師の間の権限の偏在が非効果的な協力やコミュニケーションの障壁につながり，患者ケアに悪影響を及ぼす可能性がある[18]。

### *C. 実践への具体的示唆*

本研究で明らかになった不安と期待の関連性に基づく実践的な課題として，教育的支援，組織的対応，コミュニケーションの改善という三つの観点から統合的な取り組みを推進する必要があると考えられる。

教育的支援については，看護師の不安【①急変時の実践能力不足による心配や動揺】と医師の期待【1患児の状況に合わせた予測や対応に関する知識を習得している】【2患児の状況・成長を考慮し医師の指示に基づいた処置・検査・治療の実施と環境調整ができる】の一部の側面が共通していたことから，Benner の「初心者から達人への成長モデル」に医師との協働の要素を加えた教育アプローチが求められる[19]。経験レベル別のカリキュラム構成として，初心者には基本的急変対応手順の習得，中級者には事例検討やシミュレーション訓練を通じた臨床判断力の養成，熟

練者にはチーム指揮訓練や多職種連携演習を通じたリーダーシップ強化が効果的とされる[19]。

組織的対応に関しては，不安【③不利な環境条件とその対応への心配や困難感】と期待【3緊急度に合わせたチームコミュニケーションづくり】が重視する側面が一致していることが分析から示され，単なる人員増ではなく役割の明確化と共有が重要であることが示された。情報伝達と家族の関与を重視した介入戦略を構築し，医療チーム内での効果的なコミュニケーションを促進する必要がある[20]。明確な役割分担と責任の理解に基づいた組織的な対応体制が推奨されている[21]。

コミュニケーションの改善については，不安【③不利な環境条件とその対応への心配や困難感】と医師期待【3緊急度に合わせたチームコミュニケーションづくり】の間で，重視する側面が一致していることが質的に確認された。この関連性は，緊急時におけるチームワークの強化，明確な役割分担，特定されたリーダーシップの発揮が医療の質向上に不可欠であることを示している[22]。医師と看護師がともに参加するシミュレーション訓練やその後の振り返りの場を設けることで，双方の役割認識の相違や課題を共有し，現場での連携を強化できる[23]。

以上により，教育的支援，組織的対応，コミュニケーションの改善は相互に連関しており，統合的に実践することで最大の効果が得られると考える。本研究で明らかになった看護師の不安と医師の期待の関連性から，これらの観点を取り入れた実践は，急変対応における看護師の不安軽減と医師の期待に応える実践能力の向上につながり，結果として患者の安全と医療の質向上に寄与すると考えられる。

## 本研究の限界と今後の課題

本研究で構築した概念図は，小児病棟看護師の不安と医師の期待の関連性を可視化した点で意義深い。しかし，データ収集から 10 年以上が経過し，医療技術や看護教育の変化により現在の状況とは異なる可能性がある。また，一施設での質的研究という制約から結果の一般化には限界がある。今後は多施設での量的検証や，看護師の経験年数や医師の専門性による違いなど，より詳

細な分析が必要である。特に本研究で明らかとなった看護師の実践能力への不安と，医師が期待する組織的な業務遂行能力との焦点の違いは，シミュレーション教育やチーム医療トレーニングなど，具体的な教育プログラムの開発につながる可能性を有している。これらの課題に取り組むことで，小児の急変対応における医師と看護師間の相互理解の促進と，効果的な教育支援体制の構築が期待される。

## 結　論

2 つの先行研究を統合的に分析した結果，小児病棟看護師の急変対応における不安と医師の期待との関連性が明らかになり，両者の特徴を概念図として統合的に表現した。本研究では，看護師が感じる急変時の実践能力に関する不安は，医師の期待する組織的な業務遂行能力とは異なる特徴を持ち，両者間には組織レベルと個人レベルという重視する側面の違いが存在することが示唆された。このことは，看護師の感情的要因と医師の行動的期待との間に重視する側面の違いがあることを反映している。一方，患児・家族への対応や環境条件に関する不安については，医師の期待と重視する側面が一致しており，患児・家族への対応や環境条件の整備が両職種間で共通の課題として認識されていることが明らかになった。看護師の不安と医師の期待の関連性に関する統合分析を通して得られた知見は，小児病棟における急変対応の質向上には，単に知識や技術を向上させるだけでなく，具体的には継続的な教育プログラムの実施，多職種間の相互理解を促進するための組織的な取り組み，そして家族中心ケアの実践と物理的環境整備という多角的なアプローチが不可欠であることが示された。また，本研究で得られた概念図は，看護師の不安と医師の期待の重視する側面の違いという課題に対する理解を深め，効果的な対策を講じるための基盤となることが期待される。

## 利益相反

研究者に，本研究に係わる開示すべき利益相反はない。

## IRB 承認コードと機関名

本研究は小児病棟看護師の急変対応における不安と医師の期待との関連性を明らかにするため，2 つの先行研究を質的に統合し分析した質的二次分析研究である。本研究は新たな調査を実施するものではなく, 既存の文献のみを対象としているため, 倫理審査委員会の承認は必要としない。

## 患者/参加者からの出版同意（資料の取り扱い）

本研究は公表された文献のみを用いた質的二次分析研究であり，研究参加者からの同意取得を必要としない。分析対象となる先行研究については，それぞれの研究において適切な倫理的手続きが行われていることを確認している。


## 資金提供

本研究に関する資金提供はない。

## 謝　辞

　本研究の基となる 2 つの先行研究にご協力いただいた小児病棟看護師の皆様，小児科医師の皆様に深く感謝申し上げます。また，本研究の分析過程において貴重なご意見をいただいた研究者の皆様に心より御礼申し上げます。


## 【文　献】


1. Jung M, Kim M, Lee OJ, Choi AY, Hwang T, Cho J. Characteristics and prognostic factors of previously healthy children who required respiratory support in a pediatric intensive care unit. Allergy Asthma & Respiratory Disease 2018; 6: 103-9.
2. Noreña Peña AL, Cibanal Juan L. The experience of hospitalized children regarding their interactions

with nursing professionals. Rev Lat Am Enfermagem 2011; 19: 1429-36.

3. Batista EA, Osses IE. The role of the nursing team in cardiac arrest care: Literature review. International Seven Journal of Health Research 2024; 3.

4. Tannenbaum SI, Greilich PE. The debrief imperative: building teaming competencies and team effectiveness. BMJ Qual Saf 2023; 32: 125-8.

5. Warren JL, Warren JS. The Case for Understanding Interdisciplinary Relationships in Health Care. Ochsner J 2023; 23: 94-7.

6. Essex R, Kennedy J, Miller D, Jameson J. A scoping review exploring the impact and negotiation of hierarchy in healthcare organisations. Nursing Inquiry 2023; 30: e12571.

7. Woldring JM, Gans ROB, Paans W, Luttik ML. Physicians and nurses view on their roles in communication and collaboration with families: A qualitative study. Scand J Caring Sci 2023;37:1109-22.

8. Bakunts SA. Communications of doctors and nurses. Teamwork. Probl Sotsialnoi Gig Zdravookhranenniiai Istor Med 2022; 30: 890-6.

9. Sugiura M, Morita M, Hagimoto A. Pediatric Nurses' Anxiety about Responding to Sudden Changes in Children's Condition. Journal of Japan Society of Nursing Research 2019; 42: 735-47 (in Japanese).

10. Sugiura M. Pediatricians' expectations of pediatric nurses on sudden changes in children's condition. Journal of Japanese Society of Child Health Nursing 2022; 31: 61-9 (in Japanese).

11. Ministry of Education, Culture, Sports, Science and Technology. Kango jissen noryoku ikusei no jujitsu ni muketa daigaku sotsugyauji no totatsu mokuhyo (Achievement goals at the time of university graduation for enhancing nursing practice abilities);2004 (in Japanese).<https://www.mext.go.jp/b_menu/shingi/chousa/koutou/018-15/toushin/04032601.htm>(Accessed May 5, 2025)

12. Herdman TH, Kamitsuru S. NANDA International Nursing Diagnoses: Definitions & Classification

2018-2020, 11th Edition. New York: Thieme; 2018: 403.

13. Spielberger CD: Theory and Research on Anxiety. In: Anxiety and Behavior. New York: Academic Press; 1996: 3-20.

14. Shinmura I. Kojien. 7th ed. Tokyo: Iwanami Shoten; 2018: 714 (in Japanese).

15. Miedaner F, Kuntz L, Enke C, Roth B, Nitzsche A. Exploring the differential impact of individual and organizational factors on organizational commitment of physicians and nurses BMC Health Serv Res 2018; 18: 180.

16. Nematifard T, Arsalani N, Nourozi Tabrizi K, Fallahi-Khoshknab M, Borimnejad L. Improvement of family-centered care in the pediatric rehabilitation ward: a participatory action research. Front Pediatr 2024; 12: 1325235.

17. Toida C, Morimura N. An Analysis of Stress Concerning Pediatric Emergency Care Nurses. Cureus 2022; 14: e21299.

18. Nakhaee S, Nasiri A. Inter-professional Relationships Issues among Iranian Nurses and Physicians: A Qualitative Study. Iran J Nurs Midwifery Res 2017; 22: 8-13.

19. Benner P, Ibe T, Imura M, Kamiizumi K, Niizuma K. From novice to expert: excellence and power in clinical nursing practice. Tokyo: Igaku Shoin; 2005 (in Japanese).

20. Loureiro FM, Charepe ZB. Strategies for increase satisfaction with nursing care in hospitalized children: a delphi study. Enfermeria Global 2021;20:161-75.

21. American Heart Association. Pediatric advanced life support provider manual. Tokyo: Synergy; 2021 (in Japanese).

22. Brazil V, McLean D, Lowe B, Kordich L, Cullen D, De Araujo V, Eldridge T, Purdy E. A relational approach to improving interprofessional teamwork in post-partum haemorrhage (PPH). BMC Health Serv Res 2022; 22: 1108.

23. Molina-Mula J, Gallo-Estrada J. Impact of Nurse-Patient Relationship on Quality of Care and Patient

Autonomy in Decision-Making. Int J Environ Res Public Health 2020; 17: 835.

**図のキャプション**

**図１：小児病棟看護師の急変対応における不安と医師の期待の関連性を示す概念図**

この図は，2 つの研究結果を統合して作成した概念図である。中央に配置された 4 つの円（予期，直面，対応時，対応後）は，看護師の不安の時系列的な経過を表している。図の上部には不安【③不利な環境条件とその対応への心配や困難感】，中央部には不安【②患児・家族の状態の経過から認識された危惧や動揺】，下部には不安【①急変時の実践能力不足による心配や動揺】を示した。右側には，これらの不安に対応する医師からの期待が示されており，期待【1患児の状況に合わせた予測や対応に関する知識を習得している】，期待【2患児の状況・成長を考慮し医師の指示に基づいた処置・検査・治療の実施と環境調整ができる】，期待【3緊急度に合わせたチームのコミュニケーションづくり】，期待【4家族への傾聴や児の状況説明ができる】を配置した。この図は，2 つの研究結果を統合し，看護師の不安と医師の期待で重視する側面の一致や違いを視覚的に表現したものである。特に不安【①急変時の実践能力不足による心配や動揺】については，医師の期待【1患児の状況に合わせた予測や対応に関する知識を習得している】【2患児の状況・成長を考慮し医師の指示に基づいた処置・検査・治療の実施と環境調整ができる】と重視する側面が一部共通していることが質的に確認された。この共通性は，主に患児の状況・成長に応じた実践能力**（※１）**の側面で観察された。一方，医師の指示遂行や病床の環境調整**（※２）**では重視する側面の違いが明らかとなった。

## 図表

**図１：**小児病棟看護師の急変対応における不安と医師の期待の関連性を示す概念図

**表１：**小児病棟看護師の急変対応における不安と医師の期待のコアカテゴリーの関連性

**表２：**不安①（上位カテゴリー）と期待（サブカテゴリー）の関係

**表３：**不安②（上位カテゴリー）と期待（サブカテゴリー）の関係

**表４：**不安③（上位カテゴリー）と期待（サブカテゴリー）の関係

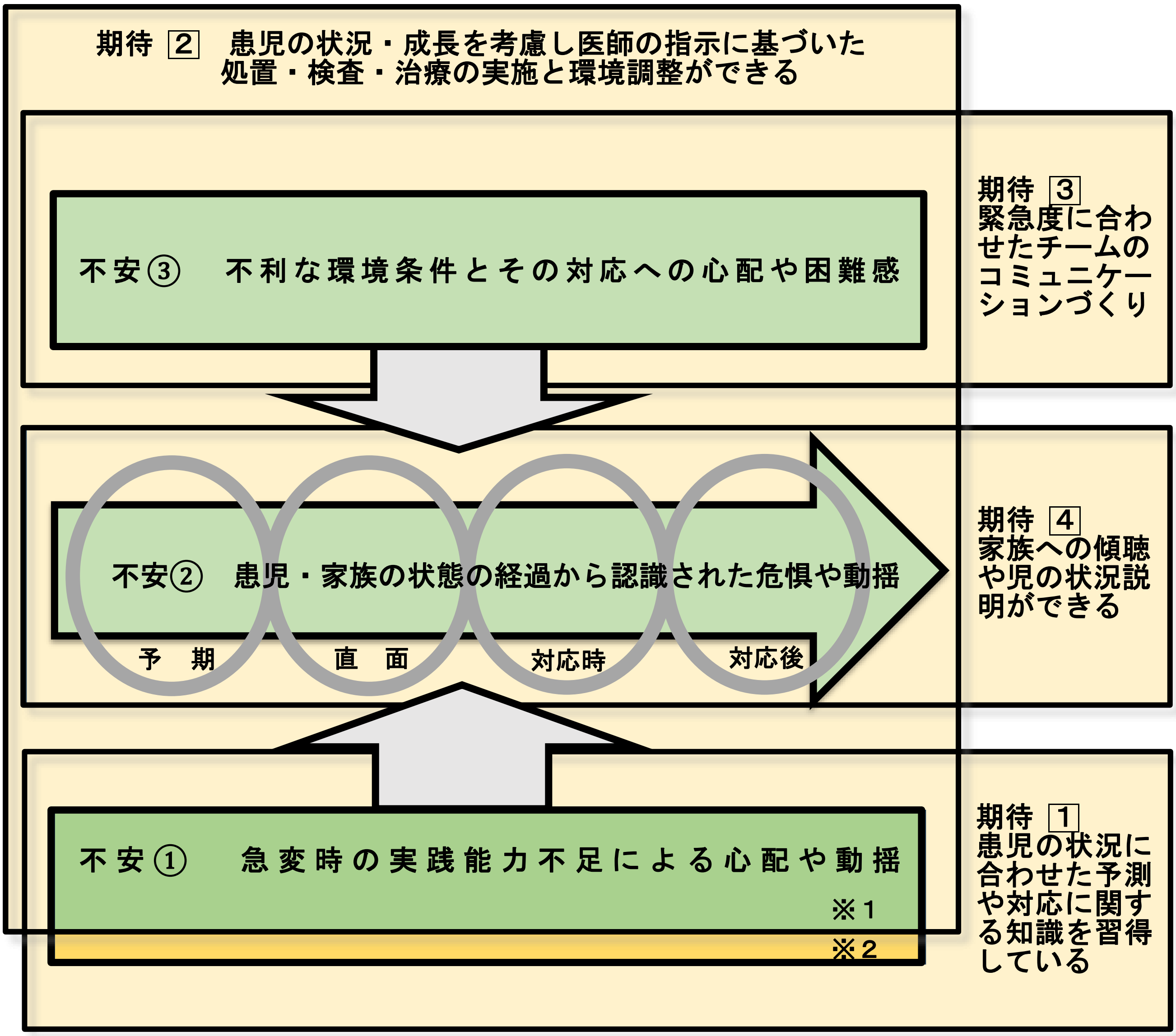


※１，２：この部分的な重なりは，主に患児の状況・成長に応じた実践能力（※１）の部分で見られるが，医師の指示遂行や病床の環境調整（※２）では重視する側面の違いが明らかとなった。

※３：図中では，不安のコアカテゴリーには丸数字（①～③），医師の期待のコアカテゴリーには四角囲み数字（1～4）を用いている。これらの記号は本文中および他の表においても一貫して使用している。

**Figure 1：小児病棟看護師の急変対応における不安と医師の期待の関連性を示す概念図**

**Tables:**

**Table 1.  小児病棟看護師の急変対応における不安と医師の期待のコアカテゴリーの関連性**

| 不安のコアカテゴリー | | 期待のコアカテゴリー | |
|---|---|---|---|
| ① | 急変時の実践能力不足による心配や動揺 | 1 | 患児の状況に合わせた予測や対応に関する知識を習得している |
| | | 2 | 患児の状況・成長を考慮し医師の指示に基づいた処置・検査・治療の実施と環境調整ができる |
| ② | 患児・家族の状態の経過から認識された危惧や動揺 | 2 | 患児の状況・成長を考慮し医師の指示に基づいた処置・検査・治療の実施と環境調整ができる |
| | | 4 | 家族への傾聴や児の状況説明ができる |
| ③ | 不利な環境条件とその対応への心配や困難感 | 2 | 患児の状況・成長を考慮し医師の指示に基づいた処置・検査・治療の実施と環境調整ができる |
| | | 3 | 緊急度に合わせたチームのコミュニケーションづくり |

表中では，不安のコアカテゴリーには丸数字（①～③），医師の期待のコアカテゴリーには四角囲み数字（1～4）を用いている。これらの記号は本文中および他の表においても一貫して使用している。

## Tables:

## Table ２． 不安①(上位カテゴリー)と期待(サブカテゴリー)の関係

| 不安のコアカテゴリー | 不安の上位カテゴリー | 期待のサブカテゴリー名 | 期待のコアカテゴリー名 |
|---|---|---|---|
| ① 急変時の実践能力不足による心配や動揺 | 1 未経験・経験不足による急変対応での動揺や困難感 | 1 患児の状態把握のための基本的知識を習得している | 1 患児の状況に合わせた予測や対応に関する知識を習得している |
| | | 2 医師の指示を予測しながら対応できる知識を習得している | |
| | | 3 患児の状況・成長に応じた物品・薬剤を使用できる状態で準備できる | 2 患児の状況・成長を考慮し医師の指示に基づいた処置・検査・治療の実施と環境調整ができる |
| | | 4 手際よく状態に応じた検査・治療・処置の介助ができる | |
| | | 5 生命維持に関する技術を実施できる | |
| | 2 児の年齢や成長に応じた急変対応に関する知識不足への心配 | 1 患児の状態把握のための基本的知識を習得している | 1 患児の状況に合わせた予測や対応に関する知識を習得している |
| | | 2 医師の指示を予測しながら対応できる知識を習得している | |
| | | 3 患児の状況・成長に応じた物品・薬剤を使用できる状態で準備できる | 2 患児の状況・成長を考慮し医師の指示に基づいた処置・検査・治療の実施と環境調整ができる |
| | | 4 手際よく状態に応じた検査・治療・処置の介助ができる | |
| | | 5 生命維持に関する技術を実施できる | |
| | 3 急変時の判断不足で責任を問われることへの危惧 | 1 患児の状態把握のための基本的知識を習得している | 1 患児の状況に合わせた予測や対応に関する知識を習得している |
| | | 2 医師の指示を予測しながら対応できる知識を習得している | |
| | | 3 患児の状況・成長に応じた物品・薬剤を使用できる状態で準備できる | 2 患児の状況・成長を考慮し医師の指示に基づいた処置・検査・治療の実施と環境調整ができる |
| | | 4 手際よく状態に応じた検査・治療・処置の介助ができる | |
| | | 5 生命維持に関する技術を実施できる | |
| | 4 児の年齢や成長に応じた急変対応における技術不足への心配や焦り | 1 患児の状態把握のための基本的知識を習得している | 1 患児の状況に合わせた予測や対応に関する知識を習得している |
| | | 2 医師の指示を予測しながら対応できる知識を習得している | |
| | | 3 患児の状況・成長に応じた物品・薬剤を使用できる状態で準備できる | 2 患児の状況・成長を考慮し医師の指示に基づいた処置・検査・治療の実施と環境調整ができる |
| | | 4 手際よく状態に応じた検査・治療・処置の介助ができる | |
| | | 5 生命維持に関する技術を実施できる | |
| | 5 感情のコントロール不足による適切な対応への困難感 | 2 医師の指示を予測しながら対応できる知識を習得している | 1 患児の状況に合わせた予測や対応に関する知識を習得している |
| | | 4 手際よく状態に応じた検査・治療・処置の介助ができる | 2 患児の状況・成長を考慮し医師の指示に基づいた処置・検査・治療の実施と環境調整ができる |
| | | 5 生命維持に関する技術を実施できる | |

**Tables:**

## Table ３．不安②(上位カテゴリー)と期待(サブカテゴリー)の関係

| 不安のコアカテゴリー | 不安の上位カテゴリー | 期待のサブカテゴリー名 | 期待のコアカテゴリー名 |
|---|---|---|---|
| ② 患児・家族の状態の経過から認識された危惧や動揺 | 6 現状から予測される急変への警戒心 | 4 手際よく状態に応じた検査・治療・処置の介助ができる | 2 患児の状況・成長を考慮し医師の指示に基づいた処置・検査・治療の実施と環境調整ができる |
| | | 5 生命維持に関する技術を実施できる | |
| | | 6 医師の指示を漏れなく遂行できる | |
| | | 7 患児周囲の病床環境を整えることができる | |
| | 7 急変に直面した時の動揺や危惧 | 5 生命維持に関する技術を実施できる | 2 患児の状況・成長を考慮し医師の指示に基づいた処置・検査・治療の実施と環境調整ができる |
| | | 7 患児周囲の病床環境を整えることができる | |
| | 8 急変が起きてしまった後悔 | 5 生命維持に関する技術を実施できる | 2 患児の状況・成長を考慮し医師の指示に基づいた処置・検査・治療の実施と環境調整ができる |
| | | 7 患児周囲の病床環境を整えることができる | |
| | 9 対応の不備による状態悪化への危惧や恐怖 | 3 患児の状況・成長に応じた物品・薬剤を使用できる状態で準備できる | 2 患児の状況・成長を考慮し医師の指示に基づいた処置・検査・治療の実施と環境調整ができる |
| | | 4 手際よく状態に応じた検査・治療・処置の介助ができる | |
| | | 5 生命維持に関する技術を実施できる | |
| | | 6 医師の指示を漏れなく遂行できる | |
| | | 7 患児周囲の病床環境を整えることができる | |
| | 10 状態が回復しないことへの焦り | 4 手際よく状態に応じた検査・治療・処置の介助ができる | 2 患児の状況・成長を考慮し医師の指示に基づいた処置・検査・治療の実施と環境調整ができる |
| | | 5 生命維持に関する技術を実施できる | |
| | | 6 医師の指示を漏れなく遂行できる | |
| | 11 自分が責任を問われることへの危惧 | 4 手際よく状態に応じた検査・治療・処置の介助ができる | 2 患児の状況・成長を考慮し医師の指示に基づいた処置・検査・治療の実施と環境調整ができる |
| | | 5 生命維持に関する技術を実施できる | |
| | | 6 医師の指示を漏れなく遂行できる | |
| | | 7 患児周囲の病床環境を整えることができる | |
| | 12 急変対応後の状態不安定による回復の見込みや生命危機に至ることへの危惧や恐怖 | 4 手際よく状態に応じた検査・治療・処置の介助ができる | 2 患児の状況・成長を考慮し医師の指示に基づいた処置・検査・治療の実施と環境調整ができる |
| | | 5 生命維持に関する技術を実施できる | |
| | | 6 医師の指示を漏れなく遂行できる | |
| | 13 想定外に重症化したことへの恐怖 | 5 生命維持に関する技術を実施できる | 2 患児の状況・成長を考慮し医師の指示に基づいた処置・検査・治療の実施と環境調整ができる |
| | | 6 医師の指示を漏れなく遂行できる | |
| | | 7 患児周囲の病床環境を整えることができる | |
| | 14 後遺症が残り責任が生じることを認識した危惧 | 5 生命維持に関する技術を実施できる | 2 患児の状況・成長を考慮し医師の指示に基づいた処置・検査・治療の実施と環境調整ができる |
| | | 6 医師の指示を漏れなく遂行できる | |
| | 15 急変時の家族の不安増強への警戒心や動揺 | 12 家族への不安軽減のための傾聴や児の状況・経過を説明できる | 4 家族への傾聴や児の状況説明ができる |
| | 16 家族の気持ちになることへの困難感 | 12 家族への不安軽減のための傾聴や児の状況・経過を説明できる | 4 家族への傾聴や児の状況説明ができる |

## Tables:

## Table ４． 不安③(上位カテゴリー)と期待(サブカテゴリー)の関係

| 不安のコアカテゴリー | 不安の上位カテゴリー | 期待のサブカテゴリー名 | 期待のコアカテゴリー名 |
|---|---|---|---|
| ③ 不利な環境条件とその対応への心配や困難感 | 17 チーム医療として医師との連携不足による急変対応への困難感や怒り | 3 患児の状況・成長に応じた物品・薬剤を使用できる状態で準備できる | 2 患児の状況・成長を考慮し医師の指示に基づいた処置・検査・治療の実施と環境調整ができる |
| | | 4 手際よく状態に応じた検査・治療・処置の介助ができる | |
| | | 5 生命維持に関する技術を実施できる | |
| | | 6 医師の指示を漏れなく遂行できる | |
| | | 7 患児周囲の病床環境を整えることができる | |
| | | 8 必要な人的環境を整え役割遂行できる | 3 緊急度に合わせたチームのコミュニケーションづくり |
| | | 9 医師・看護師間で情報共有を図る | |
| | | 10 緊急度を含めた情報伝達ができる | |
| | | 11 良い雰囲気づくりのために冷静に声をかけ合うことができる | |
| | 18 メンバー看護師の役割分担への困難感 | 3 患児の状況・成長に応じた物品・薬剤を使用できる状態で準備できる | 2 患児の状況・成長を考慮し医師の指示に基づいた処置・検査・治療の実施と環境調整ができる |
| | | 4 手際よく状態に応じた検査・治療・処置の介助ができる | |
| | | 5 生命維持に関する技術を実施できる | |
| | | 6 医師の指示を漏れなく遂行できる | |
| | | 7 患児周囲の病床環境を整えることができる | |
| | | 8 必要な人的環境を整え役割遂行できる | 3 緊急度に合わせたチームのコミュニケーションづくり |
| | | 9 医師・看護師間で情報共有を図る | |
| | | 10 緊急度を含めた情報伝達ができる | |
| | | 11 良い雰囲気づくりのために冷静に声をかけ合うことができる | |
| | 19 マンパワー不足による急変患児の対応への危惧や焦り | 3 患児の状況・成長に応じた物品・薬剤を使用できる状態で準備できる | 2 患児の状況・成長を考慮し医師の指示に基づいた処置・検査・治療の実施と環境調整ができる |
| | | 4 手際よく状態に応じた検査・治療・処置の介助ができる | |
| | | 5 生命維持に関する技術を実施できる | |
| | | 6 医師の指示を漏れなく遂行できる | |
| | | 7 患児周囲の病床環境を整えることができる | |
| | | 8 必要な人的環境を整え役割遂行できる | 3 緊急度に合わせたチームのコミュニケーションづくり |
| | | 9 医師・看護師間で情報共有を図る | |
| | | 10 緊急度を含めた情報伝達ができる | |
| | | 11 良い雰囲気づくりのために冷静に声をかけ合うことができる | |
| | 20 自分しか対応できない状況による患児の状態変化に対する困惑や焦り | 3 患児の状況・成長に応じた物品・薬剤を使用できる状態で準備できる | 2 患児の状況・成長を考慮し医師の指示に基づいた処置・検査・治療の実施と環境調整ができる |
| | | 4 手際よく状態に応じた検査・治療・処置の介助ができる | |
| | | 5 生命維持に関する技術を実施できる | |
| | | 6 医師の指示を漏れなく遂行できる | |
| | | 7 患児周囲の病床環境を整えることができる | |
| | | 8 必要な人的環境を整え役割遂行できる | 3 緊急度に合わせたチームのコミュニケーションづくり |
| | 21 マンパワー不足による別の患児への対応が不十分なことへの心配や困惑 | 8 必要な人的環境を整え役割遂行できる | 3 緊急度に合わせたチームのコミュニケーションづくり |
| | | 11 良い雰囲気づくりのために冷静に声をかけ合うことができる | |
| | 22 患児の状態に応じた物品が揃わないことによる対応遅れへの心配 | 3 患児の状況・成長に応じた物品・薬剤を使用できる状態で準備できる | 2 患児の状況・成長を考慮し医師の指示に基づいた処置・検査・治療の実施と環境調整ができる |
| | | 4 手際よく状態に応じた検査・治療・処置の介助ができる | |
| | | 5 生命維持に関する技術を実施できる | |
| | | 6 医師の指示を漏れなく遂行できる | |
| | | 7 患児周囲の病床環境を整えることができる | |
| | 23 物品の準備不足や使用不能による対応遅れへの心配や怒り | 3 患児の状況・成長に応じた物品・薬剤を使用できる状態で準備できる | 2 患児の状況・成長を考慮し医師の指示に基づいた処置・検査・治療の実施と環境調整ができる |
| | | 4 手際よく状態に応じた検査・治療・処置の介助ができる | |
| | | 5 生命維持に関する技術を実施できる | |
| | | 6 医師の指示を漏れなく遂行できる | |
| | | 7 患児周囲の病床環境を整えることができる | |
| | 24 家族不在状況による急変対応が遅れることへの心配 | 6 医師の指示を漏れなく遂行できる | 2 患児の状況・成長を考慮し医師の指示に基づいた処置・検査・治療の実施と環境調整ができる |
| | | 7 患児周囲の病床環境を整えることができる | |
| | | 8 必要な人的環境を整え役割遂行できる | 3 緊急度に合わせたチームのコミュニケーションづくり |
